# Relationship between knowledge, attitudes, and practices and COVID-19 vaccine hesitancy: A cross-sectional study in Taizhou, China

**DOI:** 10.3389/fmed.2022.770933

**Published:** 2022-08-23

**Authors:** Xiao-Qing Lin, Mei-Xian Zhang, Yan Chen, Ji-Ji Xue, He-Dan Chen, Tao-Hsin Tung, Jian-Sheng Zhu

**Affiliations:** ^1^Department of Infectious Diseases, Taizhou Hospital of Zhejiang Province, Wenzhou Medical University, Linhai, China; ^2^Evidence-Based Medicine Center, Taizhou Hospital of Zhejiang Province, Wenzhou Medical University, Linhai, China; ^3^Public Laboratory, Taizhou Hospital of Zhejiang Province, Wenzhou Medical University, Linhai, China; ^4^Department of Infectious Diseases, Taizhou Hospital, Zhejiang University, Linhai, China

**Keywords:** knowledge, attitudes, practices, KAP, COVID-19, vaccine hesitancy, China

## Abstract

**Objective:**

This study aimed to explore COVID-19 vaccine hesitancy in Chinese adults and analyzed the relationship between knowledge, attitudes, practices (KAP), and COVID-19 vaccine hesitancy.

**Methods:**

A population-based self-administered online survey was conducted in Taizhou, China to evaluate the population's hesitancy to receive COVID-19 vaccination. A total of 2.463 adults received the invitation for the survey through WeChat (A Chinese app that is used for chat, social media, and mobile payment), and 1.788 interviewees answered the structured questionnaire. The overall response rate was 72.6%.

**Results:**

Total 45.2% of people were hesitant about the COVID-19 vaccination. Using binary logistic regression analysis, we found low perception of safety (Model 3: Odds ratio = 2.977, Confidence interval: 2.237–3.963) and efficacy (Model 3: OR = 1.904, 95%CI: 1.462–2.479) of the COVID-19 vaccine in adults is the most important risk factor for COVID-19 vaccine hesitation. People who know more about COVID-19 vaccination are less hesitant (Model 2: OR = 0.967, 95% CI: 0.951–0.983). People who did not seek information independently about the COVID-19 vaccine are more likely to be skeptical (Model 4: OR = 1.300, 95% CI: 1.058–1.598, *P* = 0.013).

**Conclusion:**

In China, the population had higher levels of COVID-19 vaccine hesitation, and their knowledge of the COVID-19 vaccine, perceptions of safety and efficacy, and physical health status were significantly associated with vaccine hesitation. These results provide ideas for promoting COVID-19 vaccination and intervention and have far-reaching implications for further strengthening research on vaccine hesitancy in COVID-19 and exploring strategies for COVID-19 vaccine promotion.

## Introduction

Since the global outbreak of COVID-19, the epidemic has posed unprecedented challenges to the health care systems and economy worldwide. Developing a safe and effective vaccine and vaccination scale-up is the safest and most promising approach to effectively and sustainably prevent COVID-19 (1). Currently, 10 vaccine candidates for SARS-CoV-2 are under research or in clinical trials (2). Vaccine hesitancy is the ability to get vaccinated but refusing to receive the vaccine, delaying vaccination, or receiving the vaccine due to concerns. Vaccine hesitancy is one of the greatest threats to global health (3), and the benefits of the vaccine will be significantly hampered if there is severe hesitation to receive the COVID-19 vaccine (4). COVID-19 vaccination protects individuals from COVID-19 and establishes herd immunity and has broad benefits at the social level in terms of increased production and positive financial impact (5). Addressing COVID-19 vaccine hesitancy is significant for promoting vaccination, health protection, and social development (6).

Public knowledge, attitudes, and practices (KAP) are associated with their compliance with COVID-19 outbreak prevention and control efforts (7). COVID-19 vaccine hesitancy is a complex issue influenced by multiple factors (8). Studies on the relationship between KAP levels of COVID-19 and COVID-19 vaccine hesitancy are scarce and worthy of investigating their relationship. Therefore, we conducted a study on COVID-19 vaccine hesitancy and related factors in the Chinese population.

## Methods

### Study design and population

We conducted an anonymous online cross-sectional population-based survey *via* the WeChat-incorporated Wen-Juan-Xing platform (Changsha Ranxing Information Technology Co., Ltd., Hunan, China), the largest online survey platform in China. The target population of the survey was adults living in Mainland China. A convenient sample of 2,463 people received the invitation to the survey through WeChat, and 1,788 interviewees voluntarily answered the self-administered questionnaire by scanning the Quick Response (QR) code on their mobile phones in June 2021. A total of 2,463 adults received the invitation for the survey and 1,980 interviewees answered the structured questionnaire. A logical check was performed and outliers were eliminated before data analysis. Parents who were under 18 or over 80 years of age would be excluded. The time taken to complete the questionnaire was converted logarithmically, and if it exceeded mean ± 3SD, it was considered an outlier and was also excluded from the analysis. Finally, 1,788 questionnaires underwent data analysis, and the average time to complete the questionnaire was 876 s and the median was 753 s (ranging from 168 to 2,472 s). This study was exempted from informed consent and approved by the Ethics Committee of Taizhou Hospital of Zhejiang Province, China (Approval number: K20210520). All procedures were performed following the guidelines of our institutional ethics committee and adhered to the tenets of the Declaration of Helsinki. All participants' information was anonymous.

### Structured questionnaires

KAP surveys are commonly used to identify knowledge gaps and behavioral patterns among socio-demographic subgroups to implement effective public health interventions (9). Based on previous studies, we designed a self-administered questionnaire. The questionnaire required participants to complete closed questions with checkboxes provided for responses. The contents of the questionnaire were: (1) basic demographic information, such as age, sex, residence, education, occupation, and underlying diseases; (2) risk perception of COVID-19 was measured by a question: “How do you perceive the risk of the SARS-CoV-2?” (five items: very high, high, general, low and very low); (3) knowledge about vaccination against COVID-19 was measured by a question: “Which of the following conditions do you think is suitable for vaccination against COVID-19?” (three items: yes, no or unclear). Attitudes toward the COVID-19 vaccine were tested by the questions “How effective do you consider the COVID-19 vaccine to be when preventing novel coronavirus pneumonia?” (four items: highly effective, effective, slightly effective, or ineffective). “How safe do you consider the COVID-19 vaccine to be?” (four items: highly effective, effective, slightly effective, or ineffective) Practices were assessed by a question “Have you ever consulted the COVID-19 vaccine?” (two items: yes or no); (4) then, interviewees were asked, “Have you ever hesitated to receive vaccines against COVID-19? (whether or not you have received vaccines against COVID-19)?”. All the response options were “very hesitant,” “hesitant,” “unhesitant,” or “very unhesitant.”

### Statistical analysis

The analysis focused on the effects of the population's knowledge, attitudes, and practices on the degree of hesitation for the COVID-19 vaccine. The *t*-test and χ^2^ test were used to compare the means of continuous factors and proportions of categorical factors, respectively, to assess the difference between the hesitancy and no hesitancy groups. The potential factors associated with the population's hesitancy, such as sex, residence, education, attitudes, and practices about the COVID-19 vaccine, were initially assessed using the chi-square test. Data on age and score of knowledge about COVID-19 vaccination were continuous, expressed as mean ± standard deviation (SD), and compared the differences between the hesitancy group and the no hesitancy group using *a t*-test.

To compare the extent to which basic demographic information, level of knowledge about the COVID-19 vaccine, attitudes, and practices influenced vaccine hesitancy, variables with *P* < 0.05 in the univariate analysis were included in the model, and dominance ratios (OR) and 95% confidence intervals (CI) were calculated using binary logistic regression. Model 1 was adjusted for sex, education level, food, history of drug allergies, and suffering from chronic diseases. Additional variables were adjusted in Model 2, based on the score of knowledge about vaccination against COVID-19. Model 3 was based on perceptions of the preventive effect of the COVID-19 vaccine, perceptions of the safety of the COVID-19 vaccine. Model 4 has been following the news of the COVID-19 vaccine, COVID-19 vaccination, and proactive consultation on the COVID-19 vaccine.

Variables significant at the *P* < 0.05 level in the univariate analyses were included in the model. Data management and analysis were performed using SPSS software (version 22). A *P*-value of < 0.05 was considered to represent a statistically significant difference among the test populations.

## Results

Among 2,463 interviewees, 1,788 completed the questionnaire, and the response rate was 72.6%. 74.9% of females participated in the questionnaire, more than males, and the average age of the respondents was 41.7 ± 5.3. 58.7% of people lived in urban areas, 22.1% lived in rural areas, and the others lived in townships (Table 1). 47.7% had an education level of Junior College and above, while 29.3% had an education level of Junior Secondary and below. The largest number of people were employees and managers of enterprises, accounting for 23.2% of the total, followed by civil servants or professional technicians or servicemen (18.2%), and 15.0% were freelancers. 62.9% of people had a low-risk perception of COVID-19.

**Table 1:** Demographic characteristics of study participants (*n* = 1,788).

**Variables**	**Categories**	**Total *N* (%)/** **Mean ±SD**
Sex	Male	448 (25.1%)
	Female	1,340 (74.9%)
*Age (years)	41.7 ± 5.3	
Residence	Rural	396 (22.1%)
	Town	343 (19.2%)
	City	1,049 (58.7%)
Education level	Junior secondary and below	524 (29.3%)
	Senior secondary	412 (23.0%)
	Junior College and above	852 (47.7%)
Occupation	A civil servant or professional technician or serviceman	326 (18.2%)
	Employees and managers of enterprises	415 (23.2%)
	Workers or farmer	231 (12.9%)
	Freelancer	268 (15.0%)
	Self-employed	313 (17.5%)
	Others	235 (13.1%)
Risk perception of	High	664 (37.1%)
COVID-19	Low	1,124 (62.9%)

*Data on age were continuous, expressed as mean ± standard deviation (SD).

**Figure 1:**
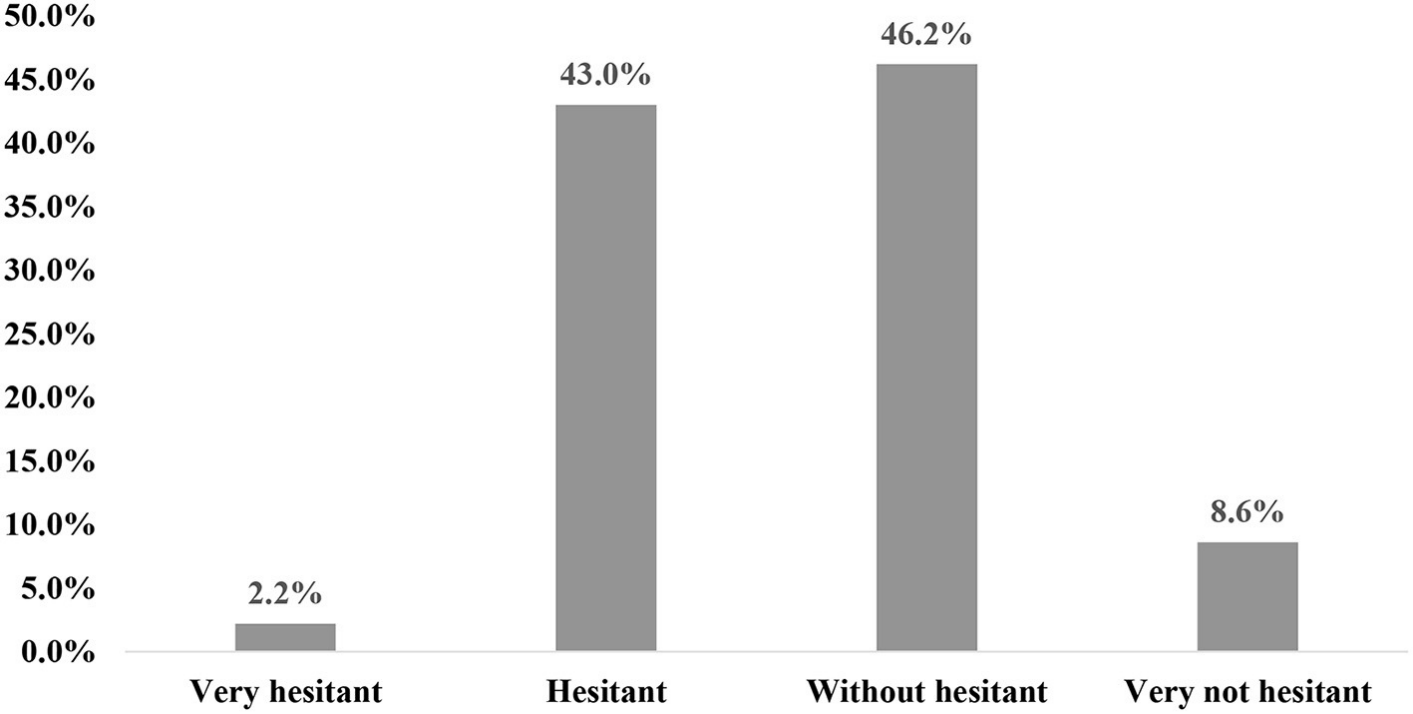
COVID-19 vaccine hesitation (*n* = 1,788).

As shown in Figure 1, among hesitant adults, 2.2% are very hesitant with the COVID-19 vaccine, 43.0% are hesitant. Among people who are unhesitating, 54.8% are unhesitating about the COVID-19 vaccine.

Table 2 shows that the population's hesitancy with COVID-19 vaccine was related to the population's knowledge, attitudes, and practices, such as a score of knowledge about COVID-19 vaccination (*t* = −2.955, *P* = 0.003), effectiveness perception of COVID-19 vaccine (χ2 = 96.984, *P* < 0.001), safety perception of COVID-19 vaccine (χ2 = 136.076, *P* < 0.001), been following the news of COVID-19 vaccine (χ2 = 17.545, *P* < 0.001), and proactive consultation on COVID-19 vaccine (χ2 = 12.541, *P* < 0.001). Table 2 shows that the basic information for adults, such as sex (χ2 = 29.699, *P* < 0.001), age (*t* = −5.088, *P* < 0.001), education level (χ2 = 8.325, *P* = 0.016). History of food and drug allergies (χ2 = 19.143, *P* < 0.001) as well as suffering from chronic diseases (χ2 = 21.939, *P* < 0.001) are related to vaccine hesitancy.

**Table 2:** Univariate analysis of factors associated with populations' COVID-19 vaccine hesitancy (*n* = 1,788).

**Variables**	**Categories**	**Hesitancy**		**No hesitancy**		***t*/χ2**	***P***
		**809**	**45.2%**	**979**	**54.8%**		
Sex						29.699	<0.001
	Male	153	34.2%	295	65.8%		
	Female	656	49.0%	684	51.0%		
Age (years)*		41.0 ± 5.1		42.3 ± 5.4		−5.088	<0.001
Residence						2.457	0.293
	Rural	166	41.9%	230	58.1%		
	Town	155	45.2%	188	488		
	City	488	46.5%	561	53.5%		
Education level						8.325^a^	**0.016**
	Junior secondary and below	210	40.1%	314	59.9%		
	Senior secondary	200	48.5%	212	51.5%		
	Junior college and above	399	46.8%	453	53.2%		
The score of knowledge about vaccination against		9.0 ± 5.9		9.9 ± 6.5		−2.955	0.003
COVID-19*					
Risk perception of COVID-19						1.080^a^	0.299
	High	311	46.8%	353	53.2%		
	Low	498	44.3%	626	55.7%		
Effectiveness perception of COVID-19 vaccine						96.984	<0.001
	High	529	38.8%	835	61.2%		
	Low	280	66.0%	114	34.0%		
							
Safety perception of COVID-19 vaccine						136.076	<0.001
	High	546	38.3%	879	61.7%		
	Low	263	72.5%	100	27.5%		
Been following the news of the COVID-19 vaccine						17.545	<0.001
	Yes	601	42.7%	807	57.3%		
	No	208	54.7%	172	45.3%		
Proactive consultation on COVID-19 vaccine						12.541^a^	<0.001
	Yes	394	49.9%	395	50.1%		
	No	415	41.5%	584	58.5%		
History of food and drug allergies						19.143	<0.001
	Yes	119	59.8%	80	40.2%		
	No	690	43.4%	899	56.6%		
Suffering from chronic diseases						21.939	<0.001
	Yes	109	61.9%	67	38.1%		
	No	700	43.4%	912	56.6%		

Data were expressed as a number followed by proportion in the parentheses within hesitancy or no hesitancy.

Data on age and score of knowledge about vaccination against COVID-19 were continuous, expressed as mean ± standard deviation (SD), and compared the differences between hesitancy group and no hesitancy group using t-test.

The results of the logistics models are shown in Table 3. There was a significant positive correlation between the population's knowledge, attitudes, practices, and COVID-19 vaccine hesitancy.

**Table 3:** Binary logistic regression analysis of factors associated with populations' COVID-19 vaccine hesitancy (*n* = 1,788).

**Variables**	**Categories**	**Model 1**		**Model 2**		**Model 3**		**Model 4**	
		***P***	**OR (95%CI)**	***P***	**OR (95%CI)**	***P***	**OR (95%CI)**	***P***	**OR (95%CI)**
Sex	Female vs. male	<0.001	1.947 (1.550–2.447)	<0.001	1.983 (1.576–2.494)	<0.001	1.744 (1.374–2.213)	<0.001	1.792 (1.410–2.278)
Education level	Junior secondary and below	1	/	1	/	1	/	1	/
	Senior secondary	0.007	1.442 (1.106–1.880)	0.002	1.515 (1.159–1.980)	0.003	1.528 (1.155–2.020)	0.003	1.541 (1.164–2.040)
	Junior college and above	0.031	1.281 (1.023–1.604)	0.001	1.471 (1.162–1.862)	0.001	1.541 (1.184–1.937)	0.001	1.511 (1.180–1.934)
History of food and drug allergies	Yes vs. no	0.001	1.687 (1.240–2.296)	0.001	1.688 (1.239–2.300)	0.003	1.629 (1.180–2.247)	0.002	1.657 (1.210–2.286)
Suffering from chronic diseases	Yes vs. no	<0.001	2.207 (1.584–3.076)	<0.001	2.304 (1.649–3.218)	<0.001	2.197 (1.556–3.101)	<0.001	2.204 (1.559–3.114)
The score of knowledge about vaccination against COVID-19*	/	/	/	<0.001	0.967 (0.951–0.983)	0.203	0.989 (0.972–1.006)	0.384	0.992 (0.975–1.010)
Effectiveness perception of COVID−19 vaccine	Low vs. high	/	/	/	/	<0.001	1.904 (1.462–2.479)	<0.001	1.870 (1.434–2.438)
Safety perception of COVID-19 vaccine	Low vs. high	/	/	/	/	<0.001	2.977 (2.237–3.963)	<0.001	2.856 (2.142–3.809)
Been following the news of COVID-19 vaccine	No vs. yes	/	/	/	/	/	/	0.112	1.230 (0.953–1.589)
Proactive consultation on COVID-19 vaccine	No vs. yes							0.013	1.300 (1.058–1.598)
**R2**		**0.057**		**0.069**		**0.162**		**0.169**	

Model 1: Demographic variables.

Model 2: Model 1 + Knowledge.

Model 3: Model 2 + Attitude.

Model 4: Model 3 + Practice.

Demographic control variables in Model 1 examine the associations between underlying health characteristics, demographics, and COVID-19 vaccine hesitancy. We found that being female (OR = 1.947, 95% CI: 1.550–2.447, *p* < 0.001), Senior Secondary (OR = 1.442, 95% CI: 1.106–1.880, *p* = 0.007), Junior College and above (OR = 1.281, 95% CI: 1.023–1.604, *p* = 0.031), having a history of food and drug allergies (OR = 1.687, 95% CI: 1.240–2.296, *P* = 0.001), and having a chronic disease (OR = 2.207, 95% CI: 1.584–3.076, *P* < 0.001) were constant factors that increased the risk of COVID-19 vaccine hesitancy.

When stratified by the score of knowledge about vaccination against COVID-19, people with higher scores are less hesitant to COVID-19 vaccination in model 2 (OR = 0.967, 95% CI: 0.951–0.983, *P* = < 0.001). Similarly, the significance of a higher risk of COVID-19 vaccine hesitation in people with lower knowledge scores can be eliminated by attitudes and practices toward the COVID-19 vaccine (Models 2 and 3).

As for attitudes toward vaccines, adults' perceptions about the preventive effects and safety of the COVID-19 vaccine have been shown to significantly influence vaccine hesitation. We found a significantly higher risk of vaccine hesitation in those who perceived low protective safety of the COVID-19 vaccine in Model 3 (OR = 2.977, 95% CI: 2.237–3.963, *P* < 0.001) and Model 4 (OR = 2.856, 95% CI: 2.142–3.809, *P* < 0.001). Similarly, Model 3 (OR = 1.904, 95% CI: 1.462–2.479, *P* < 0.001) and Model 4 (OR = 1.870, 95% CI: 1.434–2.438, *P* < 0.001) showed that vaccine hesitancy was more likely to occur among those who perceived a low effect of the COVID-19 vaccine.

The correlation between practices and vaccine hesitancy is not surprising in Model 4. People who did not proactively consult about the COVID-19 vaccine (OR = 1.300, 95% CI: 1.058–1.598, *P* = 0.013) are more likely to be hesitant.

**Figure 2:**
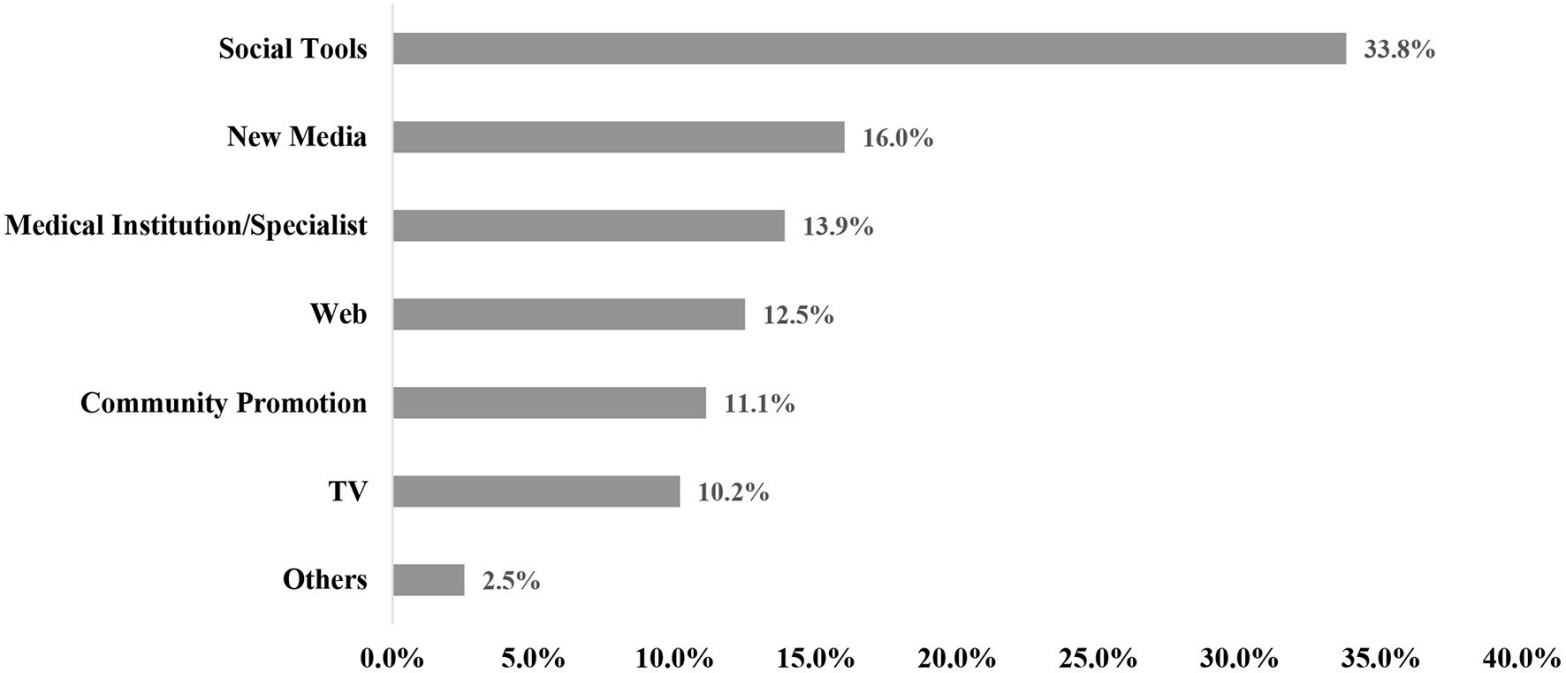
Populations' COVID-19 vaccine information sources (*n* = 1,788). Social Tools include: WeChat, QQ; New media include: Weibo, Tik Tok, Quick worker.

In our study, obtaining information through medical institutions or CDC specialists (13.9%), the community (11.1%), and social tools such as WeChat (33.8%) were the main ways for people to accessed information (Figure 2).

## Discussion

Vaccination is an important strategy to prevent and control epidemics, and vaccine hesitancy is an essential factor influencing vaccination and an important research topic in public health management. In 2012, the World Health Organization (WHO) Strategic Advisory Group of Experts on Immunization (SAGE) developed the definition: “Vaccine hesitancy is a continuum of behaviors ranging from delay in receipt to vaccination refusal.” (10). People hesitant about vaccines include those who refuse to receive vaccines, delay vaccinations, or receive vaccines but have concerns (11). Currently, the novel coronary pneumonia epidemic is still spreading globally. Accelerating COVID-19 vaccination remains the primary measure to control the epidemic, but population hesitation about the COVID-19 vaccine is still relatively common. Hesitation about the COVID-19 vaccine will affect the establishment of herd immunity for novel coronary pneumonia (12, 13).

The results of this cross-sectional study showed that the COVID-19 vaccine hesitancy rate is 45.2%. In the reported studies of COVID-19 vaccine hesitancy, public acceptance of the COVID-19 vaccine was >70% in most countries/regions (14, 15).There was a review showed COVID-19 vaccine acceptance rates ≥ 60% were seen in 72/114 countries/territories, compared to 42 countries/territories with rates between 13 and 59%. In Asia and the Pacific (*n* = 16), the highest rates were reported in Nepal and Vietnam (97%), while the lowest rate was reported in Hong Kong (42%) (16). And a study reported that 35.5% of people with vaccine hesitancy at the first round of COVID-19 vaccination in China (17).In addition, compared with hesitation for other vaccines (18, 19), there was a higher proportion of hesitation for the COVID-19 vaccine, suggesting that the Chinese population still has a certain degree of nervousness about the COVID-19 vaccine. We further investigated the risk factors for COVID-19 vaccine hesitation, including sociodemographic variables, knowledge level about the COVID-19 vaccine, attitudes toward COVID-19 vaccine safety, protective effect, and practices (20), and found that the population's perception of the COVID-19 vaccine's low perceived safety and efficacy are the main influencing factors of vaccine hesitation. A high level of knowledge about the COVID-19 vaccine and actively going for COVID-19 vaccine-related information reduces the level of vaccine hesitation. In addition, among the demographic variables, females who have a high level of education, and have a chronic disease were risk factors for vaccine hesitation.

We found that the effect of the population's knowledge, attitudes, and practices about the COVID-19 vaccine on vaccine hesitancy was significant. First, the degree of the population's perceived safety and efficacy of the vaccine was a significant predictor of vaccine hesitation, and we found that the higher the level of trust in the safety and efficacy of the COVID-19 vaccine, the lower the proportion of vaccine hesitation and the more proactive they were in getting the vaccine. Influenced by adverse vaccine safety events, people lack confidence in vaccine safety (21, 22). Some people have increased vaccine hesitancy due to the rapid spread of false information and even conspiracy theories on the Internet, receiving misinformation about vaccine safety and efficacy (23, 24). Therefore, it is critical to make credible, evidence-based information about vaccine safety and efficacy available to the population, thus improving their attitudes toward vaccines to promote vaccination. Secondly, we found that people who would actively seek advice on COVID-19 vaccine questions were more likely to receive correct and complete information about the COVID-19 vaccine and had lower levels of hesitation. Finally, our study showed that people with lower COVID-19 vaccine knowledge scores were more hesitant about the COVID-19 vaccine. It has been demonstrated that higher COVID-19 knowledge scores may be significantly associated with negative attitudes toward reducing the prevalence of COVID-19 and the likelihood of reducing potentially dangerous practices (7). Thus, individuals with higher COVID-19 vaccine knowledge scores may have higher perceptions of the safety and efficacy of the vaccine and have more positive approaches to the COVID- 19 vaccination. Thus, widespread COVID-19 vaccine knowledge can help reduce vaccine hesitation (21, 25).

Among the demographic variables, women were more hesitant than men about COVID-19 vaccination, with hesitation rates of 49.0 and 34.2%, respectively. It has been suggested that women have a more negative attitude toward COVID-19 epidemic control than men, leading to a more negative attitude toward the COVID-19 vaccine among women (7). To explore the relationship between the education level of the population and vaccine hesitancy, we found that the higher the level of education, the higher the level of vaccine hesitancy, and similar findings were found in cross-sectional studies of COVID-19 vaccine hesitancy in countries such as Canada and Spain (26). Although people with higher education may know about the vaccine (27), they may be more skeptical about the COVID-19 vaccine. Therefore, they would be more hesitant to be vaccinated. Second, the percentage of vaccine hesitancy among patients with chronic diseases was 61.9%, significantly higher than the healthy group. They may be concerned about the negative effects of COVID-19 on the underlying disease (28, 29).

It is crucial to rely on effective information dissemination methods to improve people's knowledge, attitudes, and practices regarding the COVID-19 vaccine. Different sources of vaccine information have a significant impact on vaccine hesitation (30). Therefore, medical professionals and CDC specialists can play a significant role (31).

## Limitations

The cross-sectional study was conducted at only one point in time. It did not reflect changes in the association of COVID-19 vaccine hesitancy in the population, especially when the factors influencing COVID-19 vaccine hesitancy are variable, dynamic, and multifactorial. Therefore, it is difficult to determine causality or generalize outcomes in the long term. The key to addressing such questions is to organize a series of long-term follow-up studies. This was an online questionnaire and the invitation was not sent out to an unbiased, randomly selected section of the population. As a result, people who were skeptical of vaccines were more likely to respond. And the predominance of females can be a sort of selection bias toward an overestimation of COVID-19 vaccine hesitancy considering the previous evidence of a higher prevalence of such a phenomenon among females.

## Conclusion

Vaccine hesitation is a global challenge for epidemic prevention and control, and public health management. Available studies have found that in China, the population has high levels of COVID-19 vaccine hesitation and that their knowledge of the COVID-19 vaccine, perceptions of vaccine safety and efficacy, practices, and physical health status are significantly associated with vaccine hesitation. The finding of our study may promote COVID-19 vaccination and interventions. The current situation of the novel coronary pneumonia epidemic is severe, and it is of far-reaching significance to further promote COVID-19 vaccine hesitation research and explore COVID-19 vaccine promotion strategies.

## Data availability statement

The raw data supporting the conclusions of this article will be made available by the authors, without undue reservation.

## Ethics statement

The studies involving human participants were reviewed and approved by Ethics Committee of Taizhou Hospital of Zhejiang Province (Approval number: K20210520) in China. The patients/participants provided their written informed consent to participate in this study.

## Author contributions

J-SZ and T-HT conceived the study. M-XZ, J-SZ, and T-HT designed the questionnaire. J-SZ collected the data. M-XZ was responsible for the coding of the analyses. X-QL and M-XZ analyzed and interpreted the data. X-QL wrote the first draft of the paper. J-JX, H-DC, and YC searched, sorted, and interpreted the relevant literature. All authors contributed to the article and approved the submitted version.

## Funding

This study was supported in part by the Medical and Health Science and Technology Project of Zhejiang province (2020385612) and the Science and Technology Administration Public Interest Technology Research Project of Zhejiang province (LGF19H030013).

## Conflict of interest

The authors declare that the research was conducted in the absence of any commercial or financial relationships that could be construed as a potential conflict of interest.

## Publisher's note

All claims expressed in this article are solely those of the authors and do not necessarily represent those of their affiliated organizations, or those of the publisher, the editors and the reviewers. Any product that may be evaluated in this article, or claim that may be made by its manufacturer, is not guaranteed or endorsed by the publisher.
